# Transcriptome analysis associated with polysaccharide synthesis and their antioxidant activity in *Cyclocarya paliurus* leaves of different developmental stages

**DOI:** 10.7717/peerj.11615

**Published:** 2021-06-14

**Authors:** Weida Lin, Huanwei Chen, Jianmei Wang, Yongli Zheng, Qiuwei Lu, Ziping Zhu, Na Li, Zexin Jin, Junmin Li, Hongfei Lu

**Affiliations:** 1 Zhejiang Provincial Key Laboratory of Plant Evolutionary Ecology and Conservation, Taizhou University, Taizhou, ZheJiang, China; 2 College of Life Science and Medicine, Zhejiang Sci-Tech University, Hangzhou, Zhejiang, China; 3 Forest Research Institute of Longquan City, Longquan, Zhejiang, China; 4 Zhejiang Yuanyang Agriculture Development Co. Ltd, Suichang, Zhejiang, China; 5 Zhejiang Provincial Agricultural Products Quality Safety Center, Hangzhou, Zhejiang, China

**Keywords:** *Cyclocarya paliurus*, Polysaccharide biosynthesis, Antioxidant activity, Industrial production

## Abstract

**Background:**

*Cyclocarya paliurus* (Batal.) Iljinskaja is a common endemic tree species and used as a Chinese medicine. The main active components in the leaves of this plant are polysaccharides. However, the temporal patterns of gene expression underlying the synthesis of polysaccharides in *C. paliurus* at different leaf developmental stages and its relationship with the polysaccharide content and antioxidant activities has not been reported to date.

**Methods:**

RNA-seq was used to investigate the biosynthesis pathway of polysaccharides at the four developmental stages of *C. paliurus* leaves. The content and the antioxidant activities of polysaccharides were measured with typical biochemical methods and the identified correlations were statistically evaluated.

**Results:**

Sixty-nine differentially expressed genes were found in the leaves during different developmental stages of *C. paliurus*. These are associated with glycosyltransferases and belong to 18 families. During different developmental stages of *C. paliurus*, the polysaccharide content first increased and then decreased, and the UDP-glucose 4-epimerase gene was found to be significantly positively correlated with the polysaccharide content. The clearance rates of DPPH radicals, superoxide anion radicals, hydroxyl radicals, and the reducing power of polysaccharides in the leaves of *C. paliurus* at different developmental stages showed a dose-dependent relationship with the concentration of polysaccharides.

**Conclusions:**

The smallest fully expanded leaves are suitable for high-quality tea, and leaves with sizes below the largest fully expanded leaves are suitable for industrial production of polysaccharides.

## Introduction

*Cyclocarya paliurus*, the only species of the genus *Cyclocarya* of the Juglandaceae family, is commonly known as the “sweet tea tree” in China and is traditionally used as a Chinese medicine. It grows on the cloudy highlands of southern China, including the Provinces of Jiangxi, Hunan, and Zhejiang (Xie et al., 2013; Yang et al., 2018). In China, *C. paliurus* has been traditionally used as a health food for more than 1000 years in China (Yang et al., 2018). In 2013, the leaves of *C. paliurus* have been approved as a food resource by the National Health and Family Planning Commission of China (Wang et al., 2015). The leaves of *C. paliurus* contain an abundance of natural bioactive components including steroids, saponins, polysaccharides, flavonoids, triterpenoids, and phenolic compounds (Xie et al., 2016). The main chemical components of *C. paliurus* leaves and their biological activities receive increasingly scientific attention (Cao et al., 2019; Zhai et al., 2018).

Polysaccharides extracted from *C. paliurus* leaves were found to have notable bioactivities, e.g., antioxidant (Xie et al., 2010a), hypoglycemic (Xie et al., 2006), anticancer (Xie et al., 2013), antimicrobial (Xie et al., 2012), and immunomodulatory bioactivities (Xie et al., 2014). Previous work mainly focused on extraction methods of polysaccharides from *C. paliurus* leaves (Xie et al., 2014; Xie et al., 2010a; Xie et al., 2010b). It has been shown that the applied extraction techniques significantly affect the yield, physicochemical properties, and biological activities of polysaccharides (Song et al., 2019). Different types of polysaccharides were extracted from *C. paliurus* using various methods, and the resulting monosaccharide composition and molecular weight differed (Kakar et al., 2020). For example, Xie et al. (2010b) found that *C. paliurus* polysaccharide was composed of D-xylose, L-arabinose, D-glucose, D-galactose, L-rhamnose, and D-mannose at a molar ratio of 1.00:9.67:9.65:4.96:3.29:2.70, respectively. Yang et al. (2016) reported that a different polysaccharide was composed of rhamnose, arabinose, xylose, mannose, glucose, and galactose at a molar ratio of 1.00:2.23:0.64:0.49:0.63:4.16, respectively. *C. paliurus* polysaccharide showed notable bioactivities, such as antioxidant (Xie et al., 2010a), hypoglycemic (Xie et al., 2006), anticancer (Xie et al., 2013), antimicrobial (Xie et al., 2012), and immunomodulatory activities (Xie et al., 2014). In addition, the polysaccharide content and their antioxidant activity of *C. paliurus* leaves collected from different areas and populations were studied (Liu et al., 2018; Zhou et al., 2019). Fu et al. (2015) reported the existence of seasonal and genotypic variation of the water-soluble polysaccharide content of *C. paliurus* leaves. Yang et al. (2017) studied changes of water-soluble polysaccharides of *C. paliurus* under different light regimes. Many studies have reported changes of polysaccharide contents and antioxidant activities in *C. paliurus* leaves of different planting regions, populations, and seasons. However, how the differences in polysaccharide content and antioxidant activity develop over the different developmental stages in the leaves of *C. paliurus* still remains unclear. Wang et al. (2001) reported that the water-soluble polysaccharide content of sixth-class tea leaves (i.e., fully developed leaves) was twice as high as that of first-class tea leaves (i.e., tender leaves). Petropoulos et al. (2018) reported that the antioxidant activity of *Cichorium spinosum* leaves varied at the developmental stage. At present, Chinese enterprises always collect young leaves for their good taste and shape, while mature leaves are usually collected to extract polysaccharides. Therefore, a better understanding of the content and antioxidant activity of polysaccharides in *C. paliurus* leaves at different developmental stages can help the improvement of the production processes and thus, increase economic benefits.

Recently, temporal gene expression profiles that characterize the time-dynamics of expression of specific genes related to the secondary metabolites of plants have attracted increasing scientific attention (Li et al., 2016; Liu et al., 2015; Mueller, Chiou & Leng, 2008; Shivakumar, Johnson & Zimmer, 2019; Sweetman et al., 2012; Wong et al., 2017). For example, Li et al. (2016) reported that *Camellia sinensis* leaf transcriptomes differed among three growth stages, which is also the case for differentially expressed unigenes related to metabolic pathways, biosynthesis of secondary metabolites, phenylpropanoid biosynthesis, and carbon fixation during photosynthesis. Shivakumar, Johnson & Zimmer (2019) identified differentially expressed genes (DEGs) involved in oil production using transcriptome analysis of the leaves of *Bergera koenigii* at different developmental stages. However, the temporal patterns of the gene expression of polysaccharide synthesis in *C. paliurus* at different leaf developmental stages have not been reported so far. Moreover, no systematic study has investigated the variation in the gene expression underlying polysaccharide synthesis and the relationship of these genes with the polysaccharide content and antioxidant activities in *C. paliurus* leaves during development also remain unknown.

Based on transcriptomic sequencing data, this study investigated the temporal gene expression pattern of polysaccharides biosynthesis. The content and antioxidant activity of *C. paliurus* polysaccharides at different developmental stages were also studied. Moreover, the relationship between DEGs and the content of antioxidant activity of *C. paliurus* was analyzed. These results are helpful to further develop the polysaccharide product of *C. paliurus*.

## Materials & methods

### Plant materials

*C. paliurus* leaves were collected from Zhuzhang Village (E118°48′28″, N28°5′57″), Longquan City, and Lishui City, Zhejiang Province, China, on May 1, 2018. The developmental stages of *C. paliurus* leaves have been defined by Guo et al. (2017). The F1 stage refers to the smallest fully expanded leaf, while the F4 stage refers to leaves with full leaf enlargement and full leaf thickness. F2 and F3 refer to the developmental stages between the smallest and largest leaves. According to the methods described by Li et al. (2017), different developmental stages of *C. paliurus* leaves were separately sampled at the same time on the same tree. Three plants were randomly selected and used as three biological replicates. A portion of the samples was immediately frozen in liquid nitrogen and stored at −80 °C for metabolomic and transcriptomic analyses. Another portion of the sample was brought back to the laboratory on ice and was dried at 70 °C to a constant weight, and then ground to a powder for polysaccharide extraction and analysis.

### Metabolite extraction, multiple reactions monitoring, and parameter setting

The freeze-dried leaves of *C. paliurus* were used for metabolite extraction and multiple reaction monitoring (MRM) by Metware Biotechnology Co., Ltd. (Wuhan, China) as previously described in Lin et al. (2020). Metabolites with variable importance in project (VIP) ≥1 and fold changes ≥2 or ≤0.5 were defined as significantly different in content. The MRM of each species was repeated three times.

### The biosynthesis pathway of polysaccharides in *C. paliurus* leaves

The total RNA was extracted and four cDNA libraries at four different leaf developmental stages were constructed according to the manufacturer’s protocol Paired-end sequencing of the library was performed on HiSeq XTen sequencers (Illumina, San Diego, CA, USA) by Sangon Biotech Co., Ltd. (Shanghai, China).

The raw high-throughput sequencing data or raw reads, including gene ID and sequence, were stored in the FASTA file format. The raw data were further treated and annotated according to the protocol described by Lin et al. (2020).

Bowtie2 (version 2.3.2) was used to map the quality control sequences to the assembled transcripts and RSeQC (version 2.6.1) was used for the statistical analysis of the aligned result (Lin et al., 2020). Salmon (version 0.8.2) was used to calculate the read counts and expression values of unigenes (Lin et al., 2020). Gene expression between samples were compared afterh the influence of gene lengths and sequencing discrepancies were eliminated by transcripts per million (TPM) (Lin et al., 2020). Principal component analysis (PCA) and principal co-ordinates analysis (PCoA) were performed to assess the distance and difference between samples (Chen, You & Shan, 2019). DESeq2 (version 1.12.4) was used to determine DEGs between two samples and genes were considered as significantly differentially expressed at a q-value < 0.001 and |FoldChange| > 2 (Chen, You & Shan, 2019). When the normalized expression of a gene was zero between two samples, its expression value was adjusted to 0.01 (as 0 cannot be plotted on a log plot) (Lin et al., 2020). If the normalized expression of a specific gene in two libraries was lower than 1, further differential expression analysis would be conducted without this gene (Lin et al., 2020).

Glycosyltransferase (GT) genes were identified via dbCAN HMM and sequence similarity was identified based on the CAZy database (E-Value < 1e−15, http://bcb.unl.edu/dbCAN/blast.php) (Zhang et al., 2018).

Ten glycosyltransferase genes, involved in polysaccharide biosynthesis, were randomly selected for quantitative real-time PCR (RT-qPCR) verification. These genes have gene expression (i.e., TPM value) ≥5 in at least three of the four developmental stages. The reverse transcriptase reaction of DNase-treated RNA was conducted using HiScript II reverse transcriptase according to the manufacturer’s instructions (Vazyme, Nanjing, China). Specific primers for glycosyltransferase genes were designed using Primer Premier 5.0 (Table S1). The constitutively expressed gene (the β-Actin-1 gene) was used as internal control (Zhao et al., 2018). RT-qPCR was performed using CFX Connect (BioRAD, USA). The relative expression of genes was calculated by the 2^−∆∆Ct^ method. All RT-qPCRs were performed in three biological and three technical replicates.

### Determination of the polysaccharide content

#### Extraction of polysaccharides

The leaves were dried at 50 °C for 96 h until a constant weight was obtained. Then, the leaves were ground to a fine powder and stored at room temperature until further analysis. The polysaccharides of *C. paliurus* leaves were extracted according to a previously reported method with minor modification (Fu et al., 2015). Briefly, a total of 2 g leaf powder was extracted with 80 ml of 80% ethanol at 90 °C for 60 min to remove most of the pigments, small sugar molecules, and impurities. Insoluble residues were dried and then extracted twice with 80 ml distilled water at 90 °C for 60 min. The extracts were filtered, and the filtrate was obtained after centrifugation at 4500×*g* for 15 min.

#### Content determination

The water-soluble polysaccharide content was measured using the phenol-sulphuric acid colorimetric method (Dubois et al., 1956). The absorbance was measured at 490 nm. The concentration of the water-soluble polysaccharides was quantitatively determined via the calibration curve, using glucose as standard.

### Measurement of the antioxidant activities of polysaccharides

#### Preparation of polysaccharides

The powder of the dried leaves was treated with refluxing petroleum ether twice for 2 h each to remove lipids and pigments, and the residue was air dried. Then, 20 g of samples were firstly refluxed twice with 200 mL of 80% (v/v) ethanol at 90 °C for 1 h to remove most of the small molecules. Subsequently, polysaccharides from the leaves were extracted twice with 400 mL of deionized water at 100 °C for 2 h. The extracted supernatant was collected by centrifugation at 4,000 rpm for 20 min, concentrated, and precipitated with four volumes of 95% ethanol (v/v) at 4 °C overnight, followed by centrifugation. The resultant precipitate was redissolved in distilled water, and the isolated proteins were removed approximately 10–12 times by the Sevag method (Staub, 1965), dialyzed with 14,000 Da molecular weight cutoff membrane against distilled water for 48 h, and lyophilized to obtain the partially purified *C. paliurus* polysaccharides.

#### Scavenging activity of DPPH radical

The DPPH radical scavenging capacity of polysaccharide samples was analyzed according to the method of Xie et al. Vc was used as a positive control. The ability to scavenge DPPH radical was calculated as follows: Scavenging activity (%) = 1 − (A_2_ − A_1_)/A_0_ × 100%. Here, a mixture of 0.5 mL of DPPH-ethanol solution plus 0.5 mL ethanol was used as negative control A_0_, a mixture of 0.5 mL ethanol plus 0.5 mL sample solution was used as blank A_1_, and a mixture of 0.5 mL of DPPH-ethanol solution plus 0.5 mL sample solution was used as A_2_.

#### Scavenging activity of superoxide radical

The scavenging capacities for the superoxide radical were assessed using the method previously described by Xie et al. (2016). Vc was used as positive control. The superoxide radical scavenging activity was calculated as follows: Scavenging activity (%) = 1 − A_1_/A_0_ × 100%, where A_0_ represents the change speed of absorbance of the control group in the superoxide radical generation system and A_1_ represents the change speed of absorbance of the sample.

#### Scavenging activity of hydroxyl radicals

The scavenging activity of hydroxyl radicals was assessed using the method described by Xie et al. (2016) Vc was used as positive control. The hydroxyl radicals scavenging effect was calculated using the following formula: Scavenging activity (%) = (A_2_ −A_1_)/(A_0_ − A_1_) × 100%, where A_1_ represents ultrapure water instead of sample, A_2_ represents the polysaccharide sample, and A_0_ represents ultrapure water instead of polysaccharide sample and H_2_O_2_.

#### The ferric reducing-antioxidant power

The FRAP assay was conducted according to the procedure described in the literature (He et al., 2017), with minor modification. Briefly, the FRAP reagent was prepared from 20 mmol/L iron (III) chloride solution, 10 mmol/L TPTZ solution in 40 mmol/L HCl, and 300 mmol/L sodium acetate buffer (pH 3.6) at a volume ratio of 1:1:10, respectively. The FRAP reagent was warmed in a water bath at 37 °C before use. Then, 0.1 mL of the sample and 0.4 ml ultrapure water were added to 0.5 mL of the FRAP solution and the absorbance was determined at 593 nm after 10 min. The results were expressed as VC μg/mL of polysaccharide samples.

### Statistical analysis

The data are shown as means ± standard deviation (SD) of three independent replicates. One-way analysis of variance (ANOVA) was calculated by SPSS 17 software (SPSS Inc., Chicago, IL, USA) and was used to evaluate the significance at the 0.05 level. The correlation coefficients between genes and metabolites, as well as between genes and polysaccharide content, were calculated with the corrplot package in R-3.6.1.

## Results

### Sequencing and assembly

A total of 703,862,064 reads of sequencing data, including 541,694,618 raw reads and 499,710,194 clean reads were obtained. The average Q20 and Q30 values were 98.69% and 95.23%, respectively. The average GC content was 53.08% (Table 1). A total of 296,593 unigenes were assembled, and the longest and the shortest transcript had the same length. With regard to the unigenes, 61,444 were ≥500 bp (20.72%) and 19,068 were ≥1,000 bp (6.43%). The total length of the unigenes was 127,566,956 bp, and their average length was 430.11 bp (Table 2).

**Table 1:** Summary of sequencing quality.

Sample	Raw reads	Clean reads	Clean bases	Q20 (%)	Q30 (%)	GC (%)
F1-1	60275244	57418316	8284605522	99.25%	97.14%	47.69%
F1-2	53598170	51222052	7420825268	99.28%	97.24%	48.39%
F1-3	43486608	40780112	5849968138	99.19%	96.92%	51.54%
F2-1	44202270	41685078	5992292304	99.23%	97.03%	52.97%
F2-2	61413864	58423068	8418014194	99.24%	97.10%	48.44%
F2-3	41315312	38853284	5606991844	99.23%	96.99%	54.26%
F3-1	42154028	38258314	5453521105	99.05%	96.42%	56.38%
F3-2	40111316	35177472	4690982062	97.65%	91.86%	60.67%
F3-3	34417588	30284380	4035084235	97.65%	91.82%	58.47%
F4-1	50535242	45373768	6098531357	97.93%	92.51%	49.69%
F4-2	51370720	46292002	6260829757	97.94%	92.52%	50.75%
F4-3	18814256	15942348	2274594827	98.68%	95.18%	57.74%
Summary	541694618	499710194	70386240613			

**Note:**

Q20: percentage of bases with a Phred value > 20; Q30: percentage of bases with a Phred value > 30.

**Table 2:** Length distribution of assembled transcripts and unigenes.

Nucleotide length	Transcripts	Unigenes
Total	574,747	296,593
>=500 bp	170,327	61,444
>=1,000 bp	56,093	19,068
N50	624	478
N90	240	225
Max length	76,464	76,464
Min length	201	201
Total length	289,576,116	127,566,956
Average length	503.83	430.11

### Functional annotation and classification

All 296,593 assembled unigenes were searched and annotated in NR, NT, PFAM, KOG, CDD, TrEMBL, and Swiss-Prot databases using the BLAST algorithm (E-value ≤ 1E−5). Of these unigenes, 173,809 were annotated to the NR database, accounting for 58.60% of the total. A total of 118,930 (40.10%) unigenes were annotated into the Swissprot database. The numbers of unigenes annotated to NT, PFAM, GO, KOG, CDD, and TrEMBL databases were 104,770 (35.32%), 57,649 (19.44%), 145,508 (49.06%), 64,021 (21.59%), 89,954 (30.33%), and 118,930 (40.10%), respectively. A total of 191,829 (64.68%) sequences were annotated in at least one database (Table 3).

**Table 3:** Summary of unigene annotation against publicly available databases.

Database	Number of genes	Percentage (%)
Annotated in CDD	89,954	30.33
Annotated in KOG	64,021	21.59
Annotated in NR	173,809	58.6
Annotated in NT	104,770	35.32
Annotated in PFAM	57,649	19.44
Annotated in Swissprot	118,930	40.1
Annotated in TrEMBL	168,101	56.68
Annotated in GO	145,508	49.06
Annotated in at least one database	191,829	64.68
Annotated in all database	4,897	1.65
Total genes	296,593	100

### Biosynthesis pathway of polysaccharides

**Figure 1:**
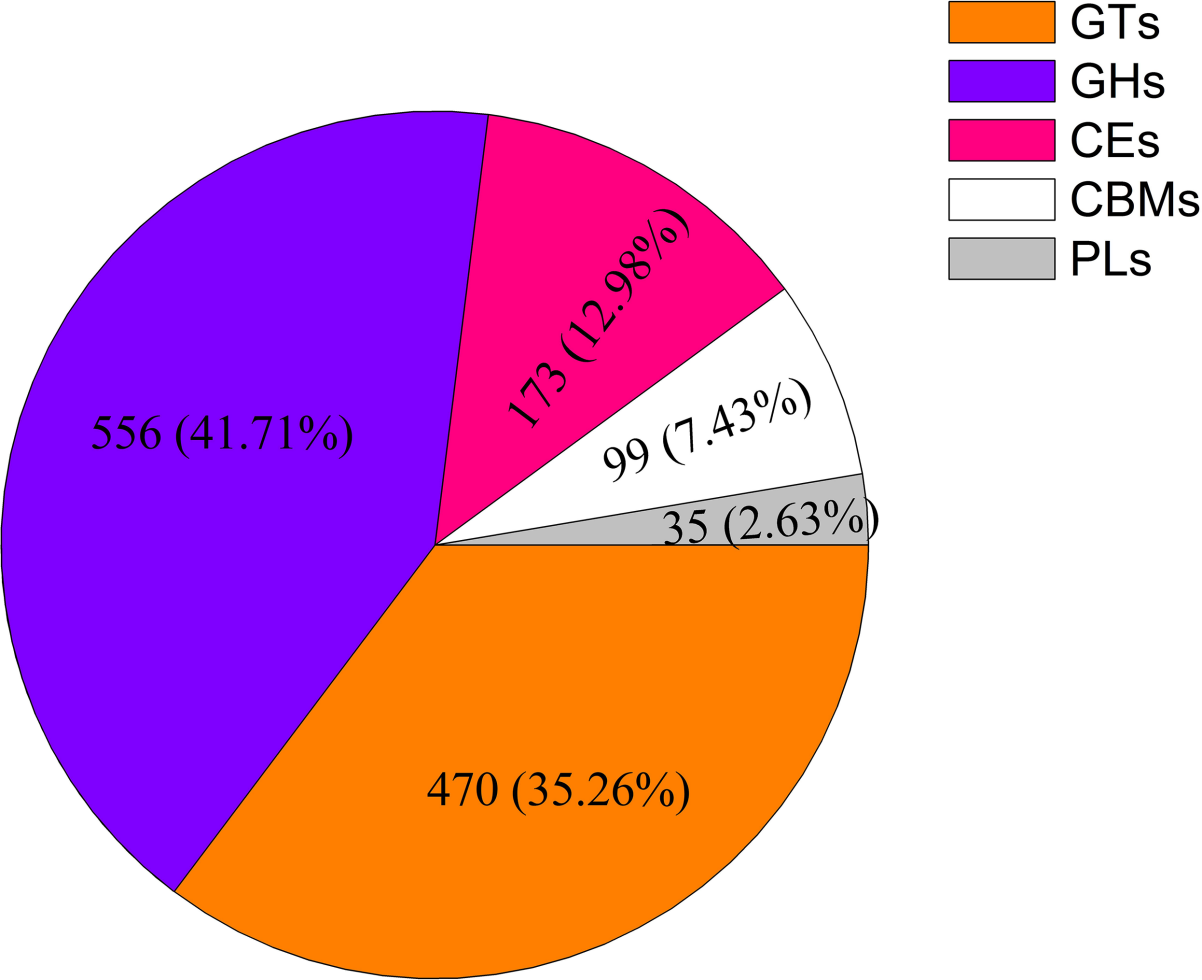
Classification and number of carbohydrate-active enzyme families in *Cyclocarya paliurus* unigenes. GT, glycosyltransferase; GH, glycoside hydrolase; CE, carbohydrate esterase; CBM, carbohydrate-binding module; PL, polysaccharide lyase.

**Figure 2:**
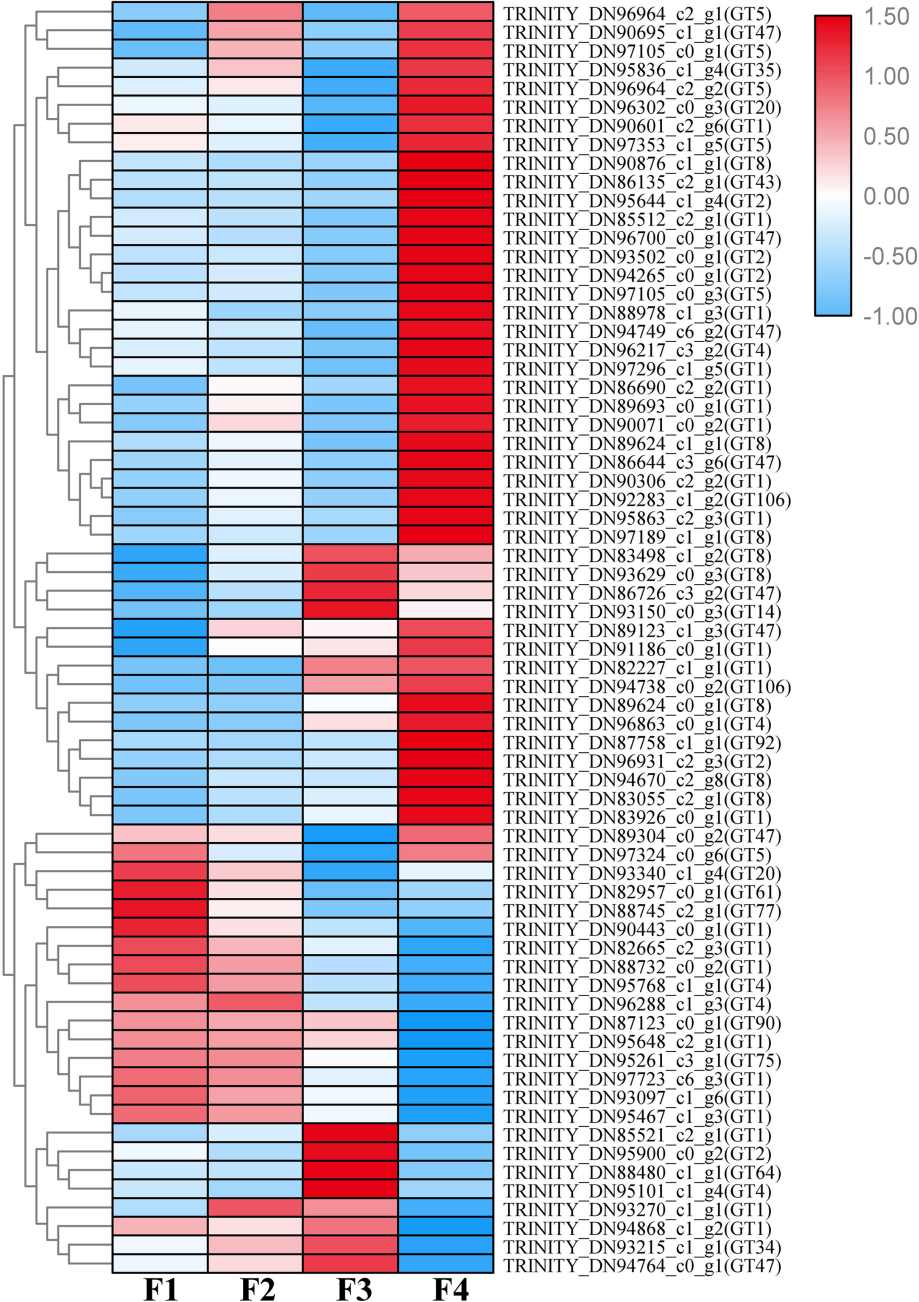
Heat map of differentially expressed glycosyltransferase genes at four different developmental stages (i.e., F1, F2, F3, and F4) of *Cyclocarya paliurus* leaves. The relative expression levels of genes are highlighted with different colors from blue (low) to red (high) across the F1, F2, F3, and F4 developmental stages.

A total of 1,333 unigenes related to the carbohydrate metabolism were identified, including 470 GT genes (which belong to 57 GT families, Table S2), 556 glycoside hydrolases, 173 carbohydrate esterases, 99 carbohydrate-binding modules, and 35 polysaccharide lyases (Fig. 1). Sixty-nine DEGs associated with GT belonging to 18 GT families were identified (Fig. 2, Table S3).

**Figure 3:**
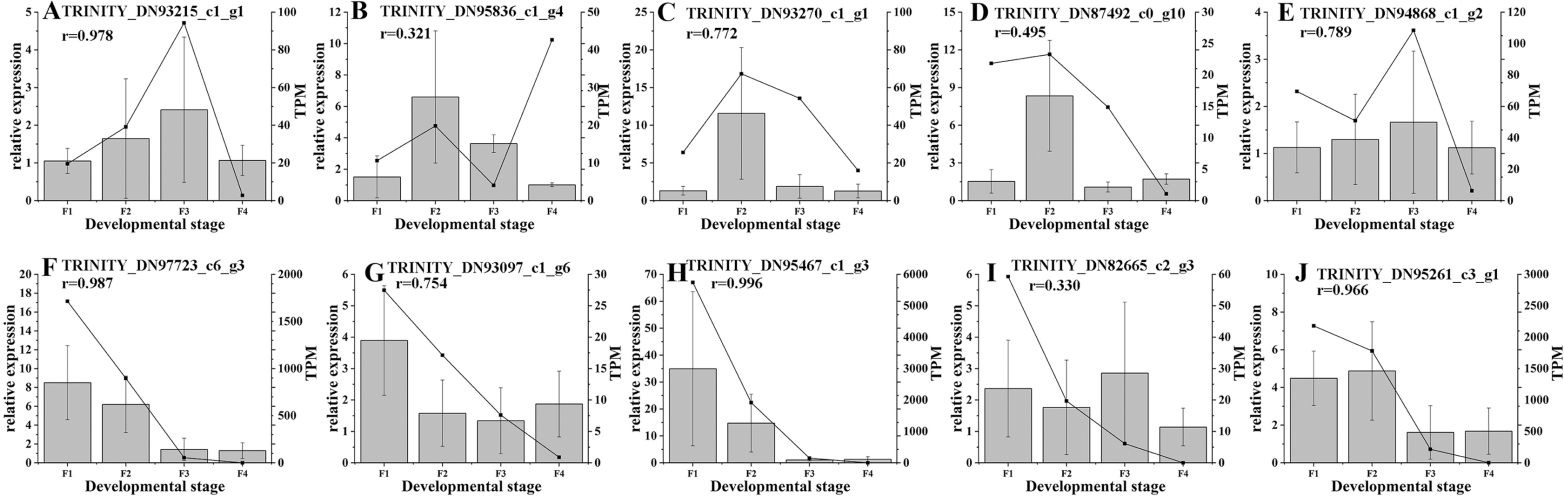
Real-time PCR validation of candidate unigenes involved in *Cyclocarya paliurus* polysaccharide biosynthesis by RNA-seq. (A–J) Respectively indicate real-time PCR validation of different genes. The histograms show the relative gene expression levels obtained via real-time PCR. The transcripts per million (TPM) of each million mapped fragment of the transcriptome are represented by line graphs. The right y-axis indicates gene expression levels calculated as TPM. The left y-axis indicates relative gene expression levels obtained by real time PCR.

**Figure 4:**
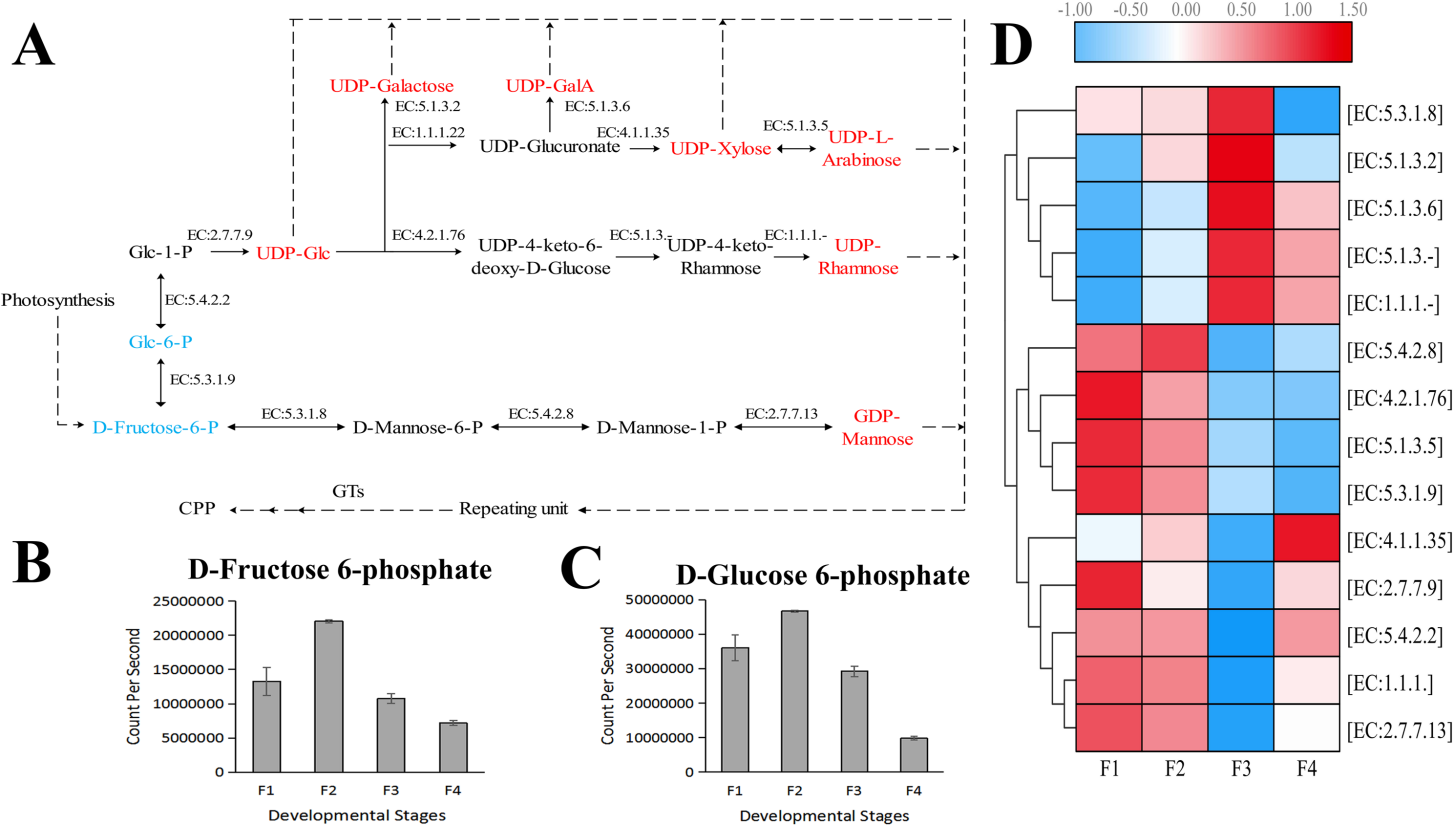
Putative biosynthesis pathway map and unigene expression pattern of *Cyclocarya paliurus* polysaccharide. (A) The pathway of *C. paliurus* polysaccharide biosynthesis was constructed based on KEGG analysis. The full names of enzymes (with EC number) are provided in Table S1. The red font represents activated monosaccharides. The text formatted in blue font represents detected metabolites. (B)–(C) The bar graph shows the contents of these two metabolites across the F1, F2, F3, and F4 developmental stages. (D) The average expression level of the enzyme-encoding unigenes at different leaf stages is indicated with a heat map. The relative expression levels of genes are highlighted with different colors from blue (low) to red (high) across the F1, F2, F3, and F4 developmental stages.

Based on the difference in expression levels of genes at four different developmental stages, 10 genes were screened for RT-qPCR (Fig. 3). All 10 selected genes had expression patterns similar to the RNA-seq data. Therefore, the data obtained in this study can be used as a tool to study the biosynthesis and metabolism-related genes of *C. paliurus*. Based on the results of transcriptome analysis and amino sugar and nucleotide sugar metabolism KEGG pathways, a pathway map for the biosynthesis of *C. paliurus* polysaccharide was proposed (Fig. 4A). In this pathway, the synthesis of *C. paliurus* polysaccharide can mainly be divided into three steps: first, formation of D-Fructose 6-phosphate; second, synthesis of multiple NDP-sugars; third, formation of polysaccharide under the action of different glycosyltransferases.

Two metabolites that are part of the polysaccharide synthesis pathway were detected in the metabolome, including D-Fructose 6-phosphate and D-Glucose 6-phosphate (Figs. 4B, 4C). The contents of D-Fructose 6-phosphate and D-Glucose 6-phosphate increased first and then decreased over the four developmental stages.

Each enzyme in the pathway was associated with several unigenes (Table S3), indicating that each enzyme in the pathway was associated with multiple coding genes. Most single genes (12 genes) were annotated as genes encoding UDP-glucuronate decarboxylase (EC: 4.1.1.35), and mannose-1-phosphate guanylyltransferase (EC: 2.7.7.13), while the second largest number of unigenes (eight genes) were annotated as UDP-glucose 4-epimerase (EC: 5.1.3.2), and phosphomannomutase (EC: 5.4.2.8) encoding genes. The third largest number of unigenes (five genes) were annotated as genes encoding UDPglucose 6-dehydrogenase (EC: 1.1.1.22), UDP-glucose 4,6-dehydratase (EC: 4.2.1.76), and UDP-glucuronate 4-epimerase (EC: 5.1.3.6). Furthermore, four unigenes were annotated as genes encoding 3,5-epimerase/4-reductase (EC:5.1.3.-), 4-reductase (EC:1.1.1.-), and mannose-6-phosphate isomerase (EC:5.3.1.8). Two unigenes were annotated as genes encoding xylan 1,4-beta-xylosidase (EC:5.1.3.5). The expressions of eight genes peaked at F1 and F2 stages while the expressions of four genes peaked at the F3 stage (Fig. 4D).

### Variation of polysaccharide contents of *C. paliurus* and its correlation with putative pathways of polysaccharides

**Figure 5:**
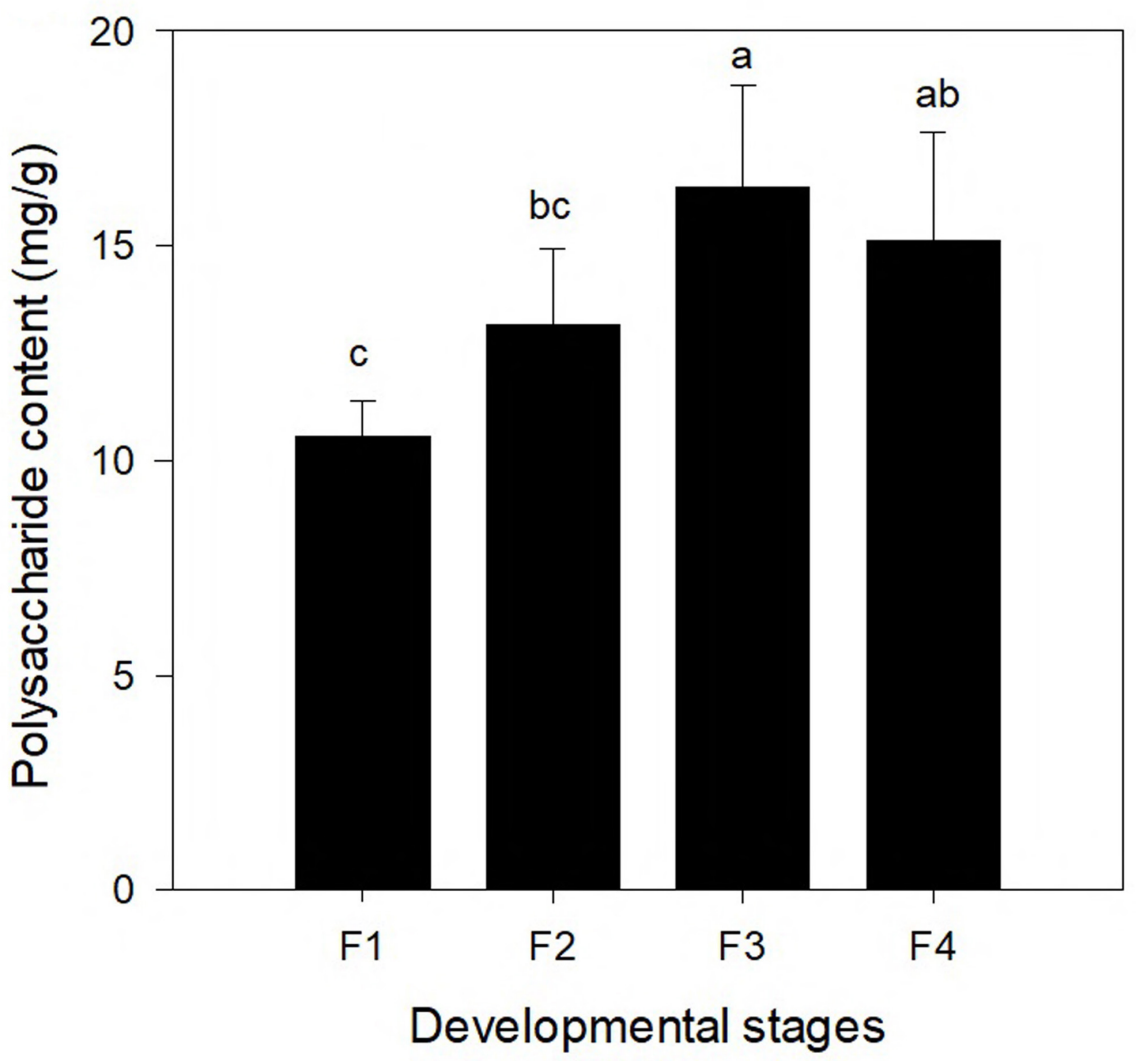
Polysaccharide content in leaves of *Cyclocarya paliurus* at four developmental stages (F1, F2, F3, and F4).

To compare the changes of polysaccharide content at different leaf development stages, the polysaccharide contents of leaves at four different developmental stages (F1, F2, F3, and F4) are shown in Fig. 5. The polysaccharide content differed significantly among the four different leaf stages. With continuing development of leaves, the polysaccharide content increased first and the highest content was found at the F3 stage (16.373 mg/g), which was followed by a decrease. The polysaccharide content in the leaves at the F4 stage was slightly lower than that at the F3 stage (but the difference was not significant).

Correlation analysis was conducted between the gene expression of genes of the polysaccharide synthesis pathway and the polysaccharide content (Table S4). One gene (TRINITY_DN83861_c1_g2, which encodes UDP-glucose 4-epimerase) was significantly positively correlated with the polysaccharide content (r = 0.037; *p* = 0.962), which encodes UDP-glucose 4-epimerase. Six genes (TRINITY_DN87479_c0_g1, TRINITY_DN92023_c2_g3, TRINITY_DN92244_c2_g3, TRINITY_DN93742_c2_g1, TRINITY_DN95705_c0_g3, and TRINITY_DN96635_c0_g1) were significantly negatively correlated with the polysaccharide content.

### Variation of the antioxidant activities of *C. paliurus* polysaccharides

**Figure 6:**
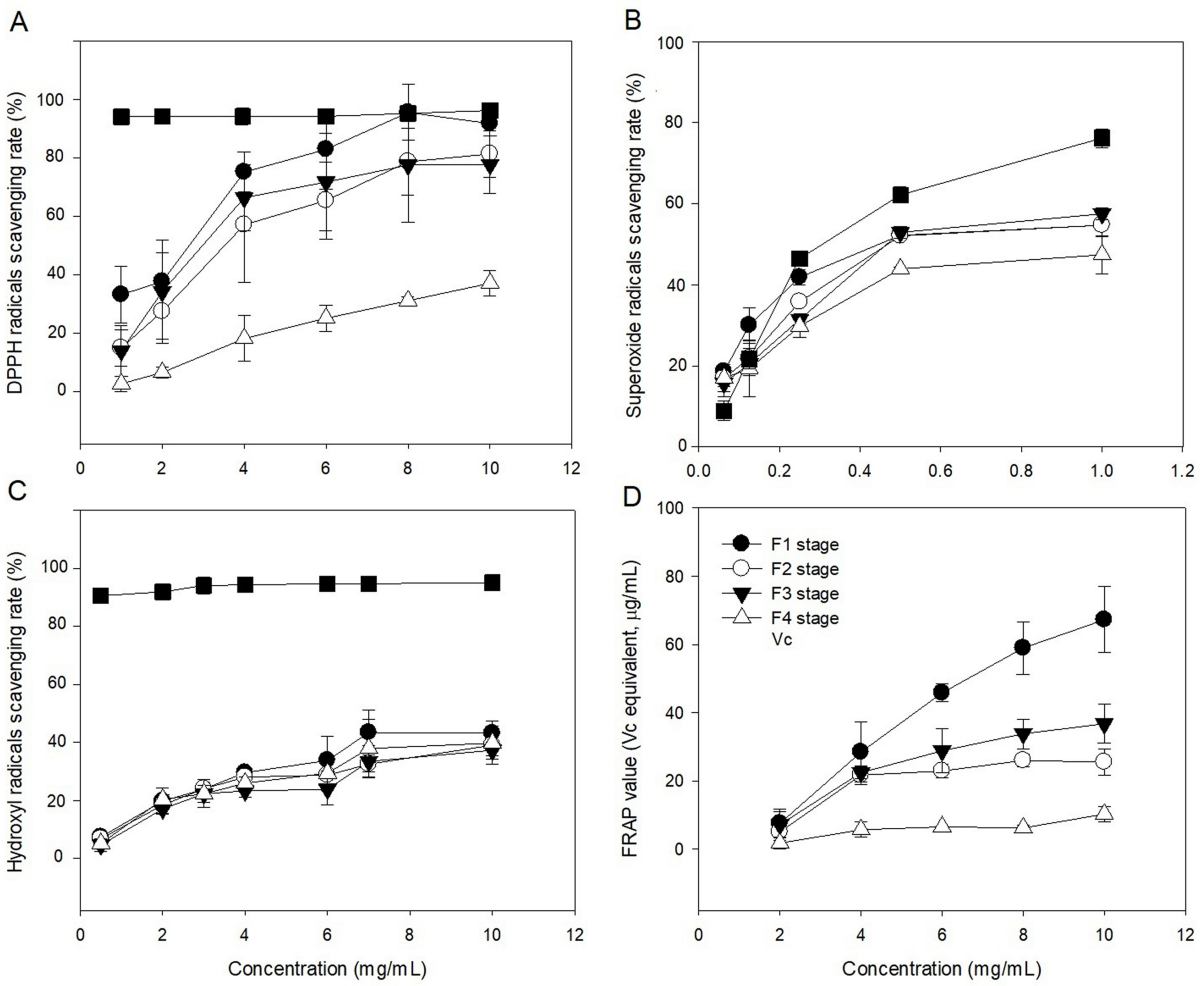
Antioxidant activities of *Cyclocarya paliurus* polysaccharides *in vitro*. (A) Scavenging activity of DPPH radicals; (B) scavenging activity of superoxide radicals; (C) scavenging activity of hydroxyl radicals; (D) reducing power.

The radical (DPPH, OH, and O^2−^) scavenging activities of polysaccharide in leaves at different developmental stages were evaluated in comparison with Vc (Figs. 6A–6C). The ability of polysaccharide and Vc to scavenge DPPH radicals was measured and the results are shown in Fig. 6A. The polysaccharide from leaves of *C. paliurus* at different developmental stages has a concentration-dependent DPPH free radicals scavenging activity (1–10 mg/mL). The F4-stage polysaccharide exhibited DPPH free radical scavenging activities below 50% within 1–10 mg/mL. With increased polysaccharide concentration, the polysaccharide extracts at the F1 stage showed similar DPPH scavenging activity with Vc between 8 and 10 mg/mL. At a concentration of 1–10 mg/mL, the scavenging rates of polysaccharide at F2 and F3 stages were lower than the rate in Vc, but higher than at the F4 stage. Among the extracted polysaccharides, the F1 stage achieved the best DPPH scavenging ability.

The abilities of polysaccharide and Vc to scavenge superoxide radicals were measured and the results are shown in Fig. 6B. The polysaccharide from leaves of *C. paliurus* at different developmental stages showed concentration-dependent superoxide free radicals scavenging activity (0.0625–1 mg/mL). The F4-stage polysaccharide exhibited superoxide free radical scavenging activities below 50% within 0.0625-1 mg/mL. With increasing polysaccharide concentration, the superoxide free radical scavenging rates of polysaccharides in the leaves at F1, F2 and F3 stages were similar, but lower than that of Vc. The superoxide anion scavenging rate of polysaccharides was lowest at the F4 stage.

The abilities of polysaccharide and Vc to scavenge hydroxyl radicals were measured and the results are shown in Fig. 6C. The polysaccharide from leaves of *C. paliurus* at different developmental stages showed concentration-dependent hydroxyl radicals scavenging activity (0.5–10 mg/mL). The hydroxyl free radical scavenging rate of polysaccharides at the four developmental stages was lower than that of Vc. At high concentrations (10 mg/ml), the scavenging activity of polysaccharides in the leaves at the developmental stages of F1, F2, F3, and F4 stages were 43.154%, 39.045%, 37.258%, and 39.757%, respectively. The scavenging rate of hydroxyl radicals by polysaccharides was highest at the F1 stage.

The polysaccharides from leaves of *C. paliurus* at different developmental stages showed concentration-dependent ferric reducing-antioxidant power (2–10 mg/mL) (Fig. 6D). With increasing polysaccharide concentration, the ferric reducing-antioxidant power of polysaccharides in the leaves had different growth rates at four developmental stages. Among which, the growth rate was highest at the F1 stage and lowest at the F4 stage. At polysaccharide concentrations of 10 mg/mL, the reducing power of polysaccharides in the leaves at the four developmental stages of F1, F2, F3, and F4 were 67.262%, 25.498%, 36.749%, and 10.251%, respectively.

## Discussion

### Transcriptome analysis associated with polysaccharide synthesis

In this study, the polysaccharide contents of *C. paliurus* leaves were found to increase first and peaked at the F3 stage. Contents at the F4 stage were lower but the difference was not statistically significance. Similar trends were reported in the other plants. For example, Robakidze & Bobkova (2003) reported that the content of water-soluble polysaccharide was higher in older needles of *Picea obovata*. Wang et al. (2001) reported that the water-soluble polysaccharide content in sixth-class tea leaves (fully developed leaves) was twice as high as that of first-class tea leaves (young leaves). In addition, the peaks of two monosaccharide precursors (D-Fructose 6-phosphate and D-Glucose 6-phosphate) appeared earlier than the polysaccharide peaks (F2 stage vs. F3 stage). This indicates that in *C. paliurus*, the synthesis of polysaccharide accompanying the consumption of photosynthate increased with the development of leaves. Compared to stage F3, the slightly lower polysaccharide content in the leaves of *C. paliurus* at the F4 stage may be the result of flower bud differentiation at that stage, which may have led to the consumption of leaf nutrients (Dickmann & Gordon, 1975).

Active nucleotide sugars (NDP-sugars) are key precursors for plant polysaccharide biosynthesis. Previous studies showed that *C. paliurus* polysaccharides are composed of various NDP sugars, such as UDP-galactose (UDP-Gal), UDP-galacturonic acid (UDP-GalA), UDP-arabinose (UDP-Ara), UDP-rhamnose (UDP-Rha), GDP-mannose (GDP-Man), UDP-xylose (UDP-Xyl), and UDP-glucose (UDP-Glc) (Wang et al., 2019; Wang et al., 2015). Among them, GDP-Man and UDP-Glc are mainly derived from fructose-6-phosphate and glucose-6-phosphate, while other NDP-sugars are mainly converted from UDP-Glc under the actions of isomerase, dehydrogenase, and decarboxylase (Yin et al., 2011). Glucose-6-phosphate has two important metabolic branches, a fructose-6-P branch and a glucose-1-P branch; therefore, enzymes that catalyze these two branch reactions are most important in the biosynthesis of polysaccharides (Zhang et al., 2019). According to the results of the present study, the gene expression of these enzymes showed different changing trends. Although the contents of glucose-6-phosphate and fructose-6-phosphate followed similar changing trends in the pathway of polysaccharide synthesis, the difference in their content still affected the resulting content of NDP sugars. The genes encoding isomerase, dehydrogenase, and decarboxylase were found to have different expression levels at different developmental stages of *C. paliurus* leaves. These differing expression levels are indicative of a tendency for specific NDP-sugar synthesis, resulting in differences in the type and proportion of NDP-sugars at different developmental stages; moreover, the structure and monosaccharide composition ratio of polysaccharides may also differ.

Transfer of sugar moieties from activated donors to specific acceptors under the formation of glycosidic bonds is a key downstream step of polysaccharide biosynthesis (Shen et al., 2017). NDP-sugars are transferred to the residues of glycoconjugates and polysaccharides via action of various GTs, where they form plant polysaccharide (Pauly et al., 2013; Wang et al., 2012). GTs (EC 2.4.x.y) are enzymes that catalyze the transfer of sugar from active donors to specific receptor molecules. GTs are involved in a variety of biological processes in plants, including plant development, signal transduction, and plant defense (Yuan et al., 2018). GTs are dominant in unigenes associated with the carbohydrate metabolism. A total of 463 and 430 GT genes identified in *A. thaliana* and *D. officinale* were assigned to 42 and 35 families, respectively (Zhang et al., 2016). The present study identified 470 GTs in *C. paliurus* that were divided into 57 GT families (Table S2). The number of GT genes and families in *C. paliurus* exceeds that in *A. thaliana* and *D. officinale* (Zhang et al., 2016). In this study, 69 DEGs associated with GT were found to belong to 18 GT families (Fig. 3), which have different functions (Zhang et al., 2016). The families with the largest number of unigenes in the GT family were GT1 (68, 14.47%), GT2 (53, 11.28%), and GT47 (41, 8.72%). Among them, the families GT106, GT7, and GT3 are specific to *C. paliurus*. These specificities may reflect the unique polysaccharide synthesis of *C. paliurus*.

Different expression of these genes at four developmental stages indicates that they may exert different roles in the process of polysaccharide biosynthesis. Up-regulated GTs are probably involved in the synthesis of soluble polysaccharides in mature leaves, while down-regulated GTs are likely mainly used to build plant cell walls and other morphological structures at the juvenile stage. Genes with similar expression trends encode multiple glycosyltransferases, suggesting that a variety of glycosyltransferases co-mediate the development of the polysaccharide biosynthesis and morphological structure. The transcripts obtained in this study provide a basis for the development of highly efficient and sustainable natural production of *C. paliurus* polysaccharides.

Correlation analysis between the accumulation of polysaccharides and biosynthesis-related gene expression identified a significant correlation between 10 unigenes and the polysaccharide content. This provides evidence for the change of polysaccharide content during the development of *C. paliurus* leaves. Yang et al. (2016) showed that the monosaccharide composition of *C. paliurus* polysaccharide was rhamnose, arabinose, xylose, mannose, glucose, and galactose at a molar ratio of 1.00:2.23:0.64:0.49:0.63:4.16, respectively. Among these, the content of galactose was highest, identifying it as an important monosaccharide component of *C. paliurus* polysaccharide. The UDP-galactose synthase gene was significantly positively correlated with the polysaccharide content, which further identifies galactose as an important component of *C. paliurus* polysaccharide. However, other nucleotide sugars did not yield a significant positive correlation, which is likely because of their low monosaccharide content in *C. paliurus* polysaccharide or because synthetic nucleotide sugars were synthesized through other synthetic pathways. The results identified the relationship between polysaccharide content and polysaccharide synthesis pathway genes, which helps to increase polysaccharide content through molecular means and thus, to increase economic benefits.

### Antioxidant activities of polysaccharide

As a stable free radical, DPPH has been widely employed to evaluate the free radical-scavenging ability of many natural compounds (Wang et al., 2014). The DPPH free radical scavenging ability of polysaccharides could be due to the presence of hydroxyls and carboxyls in the polysaccharide structure (mainly galacturonic acid). These act as hydrogen donators for scavenging the DPPH free radical, thus reducing the effects of oxidative stress (Hadidi et al., 2020). The superoxide radical is a weak oxidant, which is continuously produced by specific enzymes in biological systems and can induce the production of hydroxyl radicals and lipid peroxidation, thus damaging DNA, enzymes, and other biological molecules (Li & Shah, 2014). The possible mechanism underlying the polysaccharide scavenging of superoxide anions has been suggested to be associated with the dissociation energy of the OH bond, resulting from the number of carboxyl groups and aldehyde groups attached (Yuan et al., 2015). The hydroxyl group may subsequently provide electrons to reduce the radicals to more stable forms and/or may directly react with the free radicals to terminate the radical chain reaction thus causing the antioxidation of polysaccharides (Jing et al., 2009). The superoxide anion scavenging rate of polysaccharides was lowest at the F4 stage was lowest, which might be due to polysaccharides with lower content of carboxylic groups.

The hydroxyl radical possesses the highest activity among reactive oxygen species, enabling it to unspecifically attack adjacent biomolecules and induce oxidative damage in cells (Xu et al., 2018). The hydroxyl scavenging ability is related to the number of hydroxyl or amino groups in the polysaccharide (Guo et al., 2005). The hydroxyl radicals scavenging rate of polysaccharide were highest at the F1 stage was highest, which might be because polysaccharides had higher amino acid content at this stage. The reducing power of a compound is a useful indicator of its potential antioxidant activity (You et al., 2014). The reducing power was generally associated with the presence of reductones; therefore, polysaccharides at the F1 stage might contain higher levels of reductone, which could react with free radicals to stabilize and block radical chain reactions (Hou et al., 2012).

In the radical scavenging activity tests, the polysaccharides in leaves at different developmental stages showed different antioxidant activities. Many factors can affect the antioxidant capacity of polysaccharides, e.g., their uronic acid content, molecular weight, and monosaccharide composition (Wang et al., 2018). Several studies have postulated that the protein or peptide moiety in polysaccharides is partly responsible for their radical scavenging effect (Wang et al., 2016). Additionally, polysaccharides with lower molecular weights have more exposed reducing ends with which to accept and eliminate free radicals than polysaccharides with higher molecular weights; thus, polysaccharides with lower molecular weights might possess better antioxidant activity (Wu et al., 2020). The expression of genes in the polysaccharide biosynthesis pathway differs at different developmental stages, which may lead to different molecular weights of polysaccharides at different developmental stages, thus, in turn, leading to different antioxidant activities.

According to the results of the four antioxidant activity indexes, the polysaccharides at the F1 stage had the best antioxidant activity, followed by those at the F3 stage, while those at the F4 stage had the lowest activity. However, the polysaccharide content of leaves at the F1 stage was lowest and the biomass of leaves was also the lowest because of their small leaf size and leaf number. In summary, the leaves at the F1 stage are suitable for high-quality tea, but not suitable as raw material for polysaccharide products. Instead, the leaves at the F3 stage were more suitable for polysaccharide products.

## Conclusions

In this study, within the transcriptome database of *C. paliurus*, 1,333 carbohydrate-active enzymes were identified (including 470 glycosyltransferases) were identified. Sixty-nine DEGs associated with glycosyltransferases belong to 18 families. The UDP-glucose 4-epimerase gene was significantly positively correlated with the polysaccharide content. A pathway map for the biosynthesis of *C. paliurus* polysaccharide was proposed. The results provide a solid basis for the study of the synthesis of polysaccharides of *C. paliurus*. The polysaccharide contents and their antioxidant activities in the leaves of *C. paliurus* were assessed at four different developmental stages. The leaves at the F1 stage are suitable for high-quality tea because of the good taste and the high antioxidant ability of the contained polysaccharide. At the F3 stage, the leaves are suitable for industrial production of polysaccharide for their high polysaccharide content and relatively higher antioxidant ability of polysaccharide. These results provide clear guidance for the application of *C. paliurus* leaves for food, functional food, or medicinal applications.

## Supplemental Information

10.7717/peerj.11615/supp-1 Supplemental Information 1 Summary of sequencing quality.

10.7717/peerj.11615/supp-2 Supplemental Information 2 The category and number of GTs family in *C. paliurus*.

10.7717/peerj.11615/supp-3 Supplemental Information 3 The information of unigenes associated with putative biosynthesis pathway map of *C. paliurus* polysaccharide.

10.7717/peerj.11615/supp-4 Supplemental Information 4 Correlation coefficient of gene expression level and polysaccharide content. *Indicates significant correlation (*P*-value < 0.05, *r* > 0.9).

10.7717/peerj.11615/supp-5 Supplemental Information 5 Gene expression in polysaccharide biosynthesis pathway.

10.7717/peerj.11615/supp-6 Supplemental Information 6 The content of polysaccharides in *C. paliurus* leaves at different developmental stages.

10.7717/peerj.11615/supp-7 Supplemental Information 7 Scavenging activity (%) of DPPH radicals of *C. paliurus* polysaccharides extracted from leaves at different developmental stages.

10.7717/peerj.11615/supp-8 Supplemental Information 8 Scavenging activity (%) of superoxide radicals of *C. paliurus* polysaccharides extracted from leaves at different developmental stages.

10.7717/peerj.11615/supp-9 Supplemental Information 9 Scavenging activity (%) of hydroxyl radicals of *C. paliurus* polysaccharides extracted from leaves at different developmental stages.

10.7717/peerj.11615/supp-10 Supplemental Information 10 The reducing power (FRAP value, μg/ml) *C. paliurus* polysaccharides extracted from leaves at different developmental stages.

## Additional Information and Declarations

### Field Study Permissions

The following information was supplied relating to field study approvals (i.e., approving body and any reference numbers): Samples collection were permitted by Forest Research Institute of Longquan City, Zhejiang Province.

### DNA Deposition

The following information was supplied regarding the deposition of DNA sequences: All raw data are available at NCBI: PRJNA548403.

### Data Availability

The following information was supplied regarding data availability: The raw data are available in the Supplemental Files: Table S5 (Figure 4D), S6 (Figure 5), S7 (Figure 6A), S8 (Figure 6B), S9 (Figure 6C), S10 (Figure 6D).
