# Supplementary material for: Transcriptome analysis associated with polysaccharide synthesis and their antioxidant activity in Cyclocarya paliurus leaves of different developmental stages
Source: PeerJ. 2021 Jun 14;9:e11615. doi: 10.7717/peerj.11615 (PMC8210810; doi:10.7717/peerj.11615)
Supplement: Supplemental Information 1 [file peerj-09-11615-s001.docx]

Table S1 qRT-PCR primers used in the study

| No. | Gene ID | Sense primer（SP） | Anti-sense primer（AP） |
| --- | --- | --- | --- |
| 1 | TRINITY_DN93215_c1_g1 | 5'-AGTTCTCCAACGGTTTC-3' | 5'-GAGCCTCTGGTCCTTCCT-3' |
| 2 | TRINITY_DN95836_c1_g4 | 5'-GGCAAATGTGGAAATACG-3' | 5'-GACGAGGAAATAATCAGCAAGG-3' |
| 3 | TRINITY_DN93270_c1_g1 | 5'-TTCTTGACACGAACCCAG-3' | 5'-CCAGCCACAGTGAGATAA-3' |
| 4 | TRINITY_DN87492_c0_g10 | 5'-AAGTATGCCATTGTATCTCC-3' | 5'-GAGCACCTTACATTTAGCC-3' |
| 5 | TRINITY_DN94868_c1_g2 | 5'-GAAGGCTCACGATGGTCT-3' | 5'-TGATGCGGTGGAAATAAA-3' |
| 6 | TRINITY_DN97723_c6_g3 | 5'-AGGCTGTATTTGATTATGTC-3' | 5'-AGTTTAGGCAAGAGGTGT-3' |
| 7 | TRINITY_DN93097_c1_g6 | 5' ATGCGTGTTTCCGACTTG 3' | 5' GTGGCAGCGATATGGTGA 3' |
| 8 | TRINITY_DN95467_c1_g3 | 5' ATTACAAAGCCACCACTC 3' | 5' CCTTTCTAAACTCCGATG 3' |
| 9 | TRINITY_DN82665_c2_g3 | 5'GTGTTGTAGAGGGAGCCA 3' | 5' GGTGCAGGACGTGATTAA 3' |
| 10 | TRINITY_DN95261_c3_g1 | 5'GAAGCCAAAGCAGCGACAG 3' | 5' CCAGCCCTACCACCTCAT 3' |
| 11 | β-Actin | 5'-CTCTTCCAGCCATCCATGATCG-3' | 5'-CCACTGAGGACAATATTGCCAT-3' |
