# Supplementary material for: Transcriptome analysis associated with polysaccharide synthesis and their antioxidant activity in Cyclocarya paliurus leaves of different developmental stages
Source: PeerJ. 2021 Jun 14;9:e11615. doi: 10.7717/peerj.11615 (PMC8210810; doi:10.7717/peerj.11615)
Supplement: Supplemental Information 2 [file peerj-09-11615-s002.docx]

Table S2 The category and number of GTs family in *C. paliurus.*

| Family | Number of sequences | proportion |
| --- | --- | --- |
| GT1 | 68 | 14.47% |
| GT2 | 53 | 11.28% |
| GT3 | 3 | 0.64% |
| GT4 | 31 | 6.60% |
| GT5 | 11 | 2.34% |
| GT7 | 1 | 0.21% |
| GT8 | 38 | 8.09% |
| GT9 | 3 | 0.64% |
| GT10 | 3 | 0.64% |
| GT13 | 1 | 0.21% |
| GT14 | 15 | 3.19% |
| GT15 | 3 | 0.64% |
| GT16 | 1 | 0.21% |
| GT17 | 6 | 1.28% |
| GT19 | 2 | 0.43% |
| GT20 | 12 | 2.55% |
| GT21 | 1 | 0.21% |
| GT22 | 3 | 0.64% |
| GT24 | 2 | 0.43% |
| GT26 | 1 | 0.21% |
| GT28 | 7 | 1.49% |
| GT29 | 3 | 0.64% |
| GT30 | 2 | 0.43% |
| GT31 | 23 | 4.89% |
| GT32 | 5 | 1.06% |
| GT34 | 6 | 1.28% |
| GT35 | 2 | 0.43% |
| GT37 | 1 | 0.21% |
| GT39 | 3 | 0.64% |
| GT43 | 5 | 1.06% |
| GT47 | 41 | 8.72% |
| GT48 | 8 | 1.70% |
| GT50 | 2 | 0.43% |
| GT51 | 5 | 1.06% |
| GT56 | 1 | 0.21% |
| GT57 | 2 | 0.43% |
| GT58 | 2 | 0.43% |
| GT59 | 1 | 0.21% |
| GT61 | 2 | 0.43% |
| GT62 | 1 | 0.21% |
| GT64 | 7 | 1.49% |
| GT65 | 1 | 0.21% |
| GT66 | 2 | 0.43% |
| GT68 | 1 | 0.21% |
| GT69 | 2 | 0.43% |
| GT71 | 2 | 0.43% |
| GT75 | 7 | 1.49% |
| GT76 | 1 | 0.21% |
| GT77 | 12 | 2.55% |
| GT83 | 1 | 0.21% |
| GT87 | 1 | 0.21% |
| GT90 | 10 | 2.13% |
| GT92 | 4 | 0.85% |
| GT94 | 1 | 0.21% |
| GT95 | 4 | 0.85% |
| GT96 | 3 | 0.64% |
| GT106 | 31 | 6.60% |
