# Supplementary material for: Transcriptome analysis associated with polysaccharide synthesis and their antioxidant activity in Cyclocarya paliurus leaves of different developmental stages
Source: PeerJ. 2021 Jun 14;9:e11615. doi: 10.7717/peerj.11615 (PMC8210810; doi:10.7717/peerj.11615)
Supplement: Supplemental Information 3 [file peerj-09-11615-s003.docx]

Table S3 The information of unigenes associated with putative biosynthesis pathway map of *C. paliurus* polysaccharide.

| gene | Definition | EC number |
| --- | --- | --- |
| TRINITY_DN83861_c1_g2 | UDP-glucose 4-epimerase | EC:5.1.3.2 |
| TRINITY_DN83861_c1_g1 | UDP-glucose 4-epimerase | EC:5.1.3.2 |
| TRINITY_DN95497_c2_g1 | UDP-glucose 4-epimerase | EC:5.1.3.2 |
| TRINITY_DN98009_c0_g1 | UDP-glucose 4-epimerase | EC:5.1.3.2 |
| TRINITY_DN17106_c0_g1 | UDP-glucose 4-epimerase | EC:5.1.3.2 |
| TRINITY_DN209523_c0_g1 | UDP-glucose 4-epimerase | EC:5.1.3.2 |
| TRINITY_DN111884_c0_g1 | UDP-glucose 4-epimerase | EC:5.1.3.2 |
| TRINITY_DN92951_c3_g1 | UDP-glucose 4-epimerase | EC:5.1.3.2 |
| TRINITY_DN82527_c0_g2 | UDP-glucose 6-dehydrogenase | EC: 1.1.1.22 |
| TRINITY_DN201517_c0_g1 | UDP-glucose 6-dehydrogenase | EC: 1.1.1.22 |
| TRINITY_DN43572_c0_g1 | UDP-glucose 6-dehydrogenase | EC: 1.1.1.22 |
| TRINITY_DN93239_c0_g2 | UDP-glucose 6-dehydrogenase | EC: 1.1.1.22 |
| TRINITY_DN87479_c0_g1 | UDP-glucose 6-dehydrogenase | EC: 1.1.1.22 |
| TRINITY_DN88611_c1_g1 | UDP-glucuronate decarboxylase | EC: 4.1.1.35 |
| TRINITY_DN83085_c1_g5 | UDP-glucuronate decarboxylase | EC: 4.1.1.35 |
| TRINITY_DN82117_c0_g5 | UDP-glucuronate decarboxylase | EC: 4.1.1.35 |
| TRINITY_DN171131_c0_g1 | UDP-glucuronate decarboxylase | EC: 4.1.1.35 |
| TRINITY_DN78206_c0_g2 | UDP-glucuronate decarboxylase | EC: 4.1.1.35 |
| TRINITY_DN136939_c0_g1 | UDP-glucuronate decarboxylase | EC: 4.1.1.35 |
| TRINITY_DN158821_c0_g1 | UDP-glucuronate decarboxylase | EC: 4.1.1.35 |
| TRINITY_DN83830_c3_g2 | UDP-glucuronate decarboxylase | EC: 4.1.1.35 |
| TRINITY_DN96822_c1_g1 | UDP-glucuronate decarboxylase | EC: 4.1.1.35 |
| TRINITY_DN96822_c1_g4 | UDP-glucuronate decarboxylase | EC: 4.1.1.35 |
| TRINITY_DN94984_c0_g4 | UDP-glucuronate decarboxylase | EC: 4.1.1.35 |
| TRINITY_DN94648_c0_g4 | UDP-glucuronate decarboxylase | EC: 4.1.1.35 |
| TRINITY_DN96635_c0_g1 | xylan 1,4-beta-xylosidase | EC:5.1.3.5 |
| TRINITY_DN107831_c0_g1 | xylan 1,4-beta-xylosidase | EC:5.1.3.5 |
| TRINITY_DN138012_c0_g1 | UDP-glucose 4,6-dehydratase | EC: 4.2.1.76 |
| TRINITY_DN97446_c5_g2 | UDP-glucose 4,6-dehydratase | EC: 4.2.1.76 |
| TRINITY_DN92023_c2_g3 | UDP-glucose 4,6-dehydratase | EC: 4.2.1.76 |
| TRINITY_DN92244_c2_g3 | UDP-glucose 4,6-dehydratase | EC: 4.2.1.76 |
| TRINITY_DN98562_c0_g1 | UDP-glucose 4,6-dehydratase | EC: 4.2.1.76 |
| TRINITY_DN60941_c0_g1 | 3,5-epimerase/4-reductase | EC:5.1.3.- |
| TRINITY_DN83458_c1_g5 | 3,5-epimerase/4-reductase | EC:5.1.3.- |
| TRINITY_DN92023_c2_g7 | 3,5-epimerase/4-reductase | EC:5.1.3.- |
| TRINITY_DN61687_c0_g1 | 3,5-epimerase/4-reductase | EC:5.1.3.- |
| TRINITY_DN60941_c0_g1 | 4-reductase | EC:1.1.1.- |
| TRINITY_DN83458_c1_g5 | 4-reductase | EC:1.1.1.- |
| TRINITY_DN92023_c2_g7 | 4-reductase | EC:1.1.1.- |
| TRINITY_DN61687_c0_g1 | 4-reductase | EC:1.1.1.- |
| TRINITY_DN91459_c1_g3 | UDP-glucuronate 4-epimerase | EC: 5.1.3.6 |
| TRINITY_DN91806_c0_g3 | UDP-glucuronate 4-epimerase | EC: 5.1.3.6 |
| TRINITY_DN95773_c1_g1 | UDP-glucuronate 4-epimerase | EC: 5.1.3.6 |
| TRINITY_DN91060_c3_g5 | UDP-glucuronate 4-epimerase | EC: 5.1.3.6 |
| TRINITY_DN90463_c2_g1 | UDP-glucuronate 4-epimerase | EC: 5.1.3.6 |
| TRINITY_DN202488_c0_g1 | phosphomannomutase | EC: 5.4.2.8 |
| TRINITY_DN77554_c0_g2 | phosphomannomutase | EC: 5.4.2.8 |
| TRINITY_DN168623_c0_g1 | phosphomannomutase | EC: 5.4.2.8 |
| TRINITY_DN89461_c0_g3 | phosphomannomutase | EC: 5.4.2.8 |
| TRINITY_DN193194_c0_g1 | phosphomannomutase | EC: 5.4.2.8 |
| TRINITY_DN89461_c0_g1 | phosphomannomutase | EC: 5.4.2.8 |
| TRINITY_DN174495_c0_g1 | phosphomannomutase | EC: 5.4.2.8 |
| TRINITY_DN63399_c0_g1 | phosphomannomutase | EC: 5.4.2.8 |
| TRINITY_DN150323_c0_g1 | guanylyltransferase | EC: 2.7.7.13 |
| TRINITY_DN92316_c0_g10 | guanylyltransferase | EC: 2.7.7.13 |
| TRINITY_DN135715_c0_g1 | guanylyltransferase | EC: 2.7.7.13 |
| TRINITY_DN23511_c0_g1 | guanylyltransferase | EC: 2.7.7.13 |
| TRINITY_DN89112_c2_g1 | guanylyltransferase | EC: 2.7.7.13 |
| TRINITY_DN87865_c1_g5 | guanylyltransferase | EC: 2.7.7.13 |
| TRINITY_DN89112_c2_g6 | guanylyltransferase | EC: 2.7.7.13 |
| TRINITY_DN125684_c0_g1 | guanylyltransferase | EC: 2.7.7.13 |
| TRINITY_DN69133_c0_g1 | guanylyltransferase | EC: 2.7.7.13 |
| TRINITY_DN55690_c0_g1 | guanylyltransferase | EC: 2.7.7.13 |
| TRINITY_DN92316_c0_g1 | guanylyltransferase | EC: 2.7.7.13 |
| TRINITY_DN95705_c0_g3 | guanylyltransferase | EC: 2.7.7.13 |
| TRINITY_DN76754_c0_g1 | mannose-6-phosphate isomerase | EC:5.3.1.8 |
| TRINITY_DN75066_c0_g1 | mannose-6-phosphate isomerase | EC:5.3.1.8 |
| TRINITY_DN95388_c1_g6 | mannose-6-phosphate isomerase | EC:5.3.1.8 |
| TRINITY_DN34736_c0_g1 | mannose-6-phosphate isomerase | EC:5.3.1.8 |
| TRINITY_DN128421_c0_g1 | Uridine-diphosphate glucose pyrophosphorylase | EC:2.7.7.9 |
| TRINITY_DN44878_c0_g1 | Uridine-diphosphate glucose pyrophosphorylase | EC:2.7.7.9 |
| TRINITY_DN83373_c2_g5 | Uridine-diphosphate glucose pyrophosphorylase | EC:2.7.7.9 |
| TRINITY_DN80549_c0_g2 | Uridine-diphosphate glucose pyrophosphorylase | EC:2.7.7.9 |
| TRINITY_DN137688_c0_g1 | Uridine-diphosphate glucose pyrophosphorylase | EC:2.7.7.9 |
| TRINITY_DN84933_c2_g10 | Uridine-diphosphate glucose pyrophosphorylase | EC:2.7.7.9 |
| TRINITY_DN84933_c2_g11 | Uridine-diphosphate glucose pyrophosphorylase | EC:2.7.7.9 |
| TRINITY_DN84933_c2_g12 | Uridine-diphosphate glucose pyrophosphorylase | EC:2.7.7.9 |
| TRINITY_DN28124_c0_g1 | Uridine-diphosphate glucose pyrophosphorylase | EC:2.7.7.9 |
| TRINITY_DN84933_c2_g1 | Uridine-diphosphate glucose pyrophosphorylase | EC:2.7.7.9 |
| TRINITY_DN94917_c0_g6 | Uridine-diphosphate glucose pyrophosphorylase | EC:2.7.7.9 |
| TRINITY_DN96447_c1_g1 | Phosphoglucomutase | EC:5.4.2.2 |
| TRINITY_DN41566_c0_g1 | Phosphoglucomutase | EC:5.4.2.2 |
| TRINITY_DN94787_c0_g1 | Phosphoglucomutase | EC:5.4.2.2 |
| TRINITY_DN76734_c0_g1 | Phosphoglucomutase | EC:5.4.2.2 |
| TRINITY_DN119039_c0_g1 | Phosphoglucomutase | EC:5.4.2.2 |
| TRINITY_DN35514_c0_g1 | Phosphoglucomutase | EC:5.4.2.2 |
| TRINITY_DN81021_c4_g6 | Phosphoglucomutase | EC:5.4.2.2 |
| TRINITY_DN61651_c0_g2 | Glucose-6-phosphate isomerase | EC:5.3.1.9 |
| TRINITY_DN96738_c1_g3 | Glucose-7-phosphate isomerase | EC:5.3.1.9 |
| TRINITY_DN91914_c1_g3 | Glucose-8-phosphate isomerase | EC:5.3.1.9 |
| TRINITY_DN82144_c4_g4 | Glucose-9-phosphate isomerase | EC:5.3.1.9 |
| TRINITY_DN93347_c3_g1 | Glucose-10-phosphate isomerase | EC:5.3.1.9 |
| TRINITY_DN95019_c0_g3 | Glucose-11-phosphate isomerase | EC:5.3.1.9 |
| TRINITY_DN192700_c0_g1 | Glucose-12-phosphate isomerase | EC:5.3.1.9 |
| TRINITY_DN82664_c1_g1 | Glucose-13-phosphate isomerase | EC:5.3.1.9 |
| TRINITY_DN71018_c2_g1 | Glucose-14-phosphate isomerase | EC:5.3.1.9 |
|  |  |  |
|  |  |  |
|  |  |  |
|  |  |  |
|  |  |  |
|  |  |  |
|  |  |  |
|  |  |  |
|  |  |  |
|  |  |  |
|  |  |  |
|  |  |  |
|  |  |  |
|  |  |  |
|  |  |  |
|  |  |  |
|  |  |  |
