# Supplementary material for: Transcriptome analysis associated with polysaccharide synthesis and their antioxidant activity in Cyclocarya paliurus leaves of different developmental stages
Source: PeerJ. 2021 Jun 14;9:e11615. doi: 10.7717/peerj.11615 (PMC8210810; doi:10.7717/peerj.11615)
Supplement: Supplemental Information 4 — *Indicates significant correlation (P-value < 0.05, r > 0.9). [file peerj-09-11615-s004.docx]

**Table S7 Correlation coefficient of gene expression level and polysaccharide content.** * indicates significant correlation (P-value<0.05, r>0.9).

| GENE1 | P-value | Correlation coefficient |
| --- | --- | --- |
| TRINITY_DN193194_c0_g1 | 0.324717387 | 0.675282613 |
| TRINITY_DN82117_c0_g5 | 0.124574363 | -0.875425638 |
| TRINITY_DN82527_c0_g2 | 0.570461798 | -0.429538202 |
| TRINITY_DN83085_c1_g5 | 0.6939466 | 0.3060534 |
| TRINITY_DN83458_c1_g5 | 0.093277491 | 0.906722509 |
| TRINITY_DN83458_c1_g5 | 0.093277491 | 0.906722509 |
| TRINITY_DN83830_c3_g2 | 0.783331145 | 0.216668855 |
| TRINITY_DN83861_c1_g2 | 0.037060034 | 0.962939966* |
| TRINITY_DN84086_c1_g1 | 0.113620982 | -0.886379018 |
| TRINITY_DN87479_c0_g1 | 0.044020676 | -0.955979324* |
| TRINITY_DN87865_c1_g5 | 0.066765597 | -0.933234403 |
| TRINITY_DN88611_c1_g1 | 0.392859061 | -0.607140939 |
| TRINITY_DN89363_c3_g4 | 0.176047859 | 0.823952141 |
| TRINITY_DN89461_c0_g1 | 0.483495816 | -0.516504184 |
| TRINITY_DN89461_c0_g3 | 0.472952659 | -0.527047341 |
| TRINITY_DN89907_c1_g2 | 0.905612004 | -0.094387996 |
| TRINITY_DN90463_c2_g1 | 0.17264225 | 0.82735775 |
| TRINITY_DN91060_c3_g5 | 0.514285657 | 0.485714343 |
| TRINITY_DN91806_c0_g3 | 0.476503315 | 0.523496685 |
| TRINITY_DN92023_c2_g3 | 0.022907056 | -0.977092944* |
| TRINITY_DN92244_c2_g3 | 0.025605009 | -0.974394991* |
| TRINITY_DN92316_c0_g1 | 0.437443249 | -0.562556751 |
| TRINITY_DN92951_c3_g1 | 0.513929676 | 0.486070324 |
| TRINITY_DN93239_c0_g2 | 0.321710085 | -0.678289915 |
| TRINITY_DN93742_c2_g1 | 0.023770593 | -0.976229407* |
| TRINITY_DN94648_c0_g4 | 0.759414895 | 0.240585105 |
| TRINITY_DN95388_c1_g6 | 0.589257137 | 0.410742863 |
| TRINITY_DN95705_c0_g3 | 0.01365868 | -0.98634132* |
| TRINITY_DN95773_c1_g1 | 0.152658823 | 0.847341177 |
| TRINITY_DN96635_c0_g1 | 0.043132876 | -0.956867124* |
| TRINITY_DN96822_c1_g1 | 0.876651733 | 0.123348267 |
| TRINITY_DN96822_c1_g4 | 0.167000819 | -0.832999181 |
| TRINITY_DN97446_c5_g2 | 0.094584996 | -0.905415004 |
| TRINITY_DN97571_c1_g4 | 0.453946954 | 0.546053046 |
